# Genistein Stimulates Jejunum Chloride Secretion via an Akt-Mediated Pathway in Intact Female Mice

**DOI:** 10.1159/000373953

**Published:** 2015-02-12

**Authors:** Lana Leung, Ashesh Bhakta, Katherine Cotangco, Layla Al-Nakkash

**Affiliations:** Department of Physiology, Midwestern University, Glendale, AZ, USA

**Keywords:** Genistein, Estradiol, Chloride secretion, Jejunum, Intestine, Signaling pathways, Intact mice, Ovariectomized mice

## Abstract

**Background/Aims:**

We have previously shown that daily subcutaneous injections with the naturally occurring phytoestrogen genistein (600 mg genistein/kg body weight/day, 600G) results in a significantly increased basal intestinal chloride, Cl^−^, secretion (I_sc_, a measure of transepithelial secretion) in intact C57BL/6J female mice after 1-week of treatment, compared to controls (DMSO vehicle injected). Removal of endogenous estrogen via ovariectomy (OVX) had no effect on the 600G-mediated increase in basal I_sc_.

**Methods:**

Given the estrogen-like characteristics of genistein, we compared the effects of daily estradiol (E2) injections (10 mg E2/kg body weight/day, 10E2) on basal I_sc_ in intact and OVX mice. In intact mice, 10E2 was without effect on basal I_sc_, however, in OVX mice, 10E2 significantly increased basal I_sc_ (mimicked 600G). The goal of the current study was to characterize the intracellular signaling pathways responsible for mediating 600G- or 10E2-stimulated increases in basal I_sc_ in intact female or OVX mice.

**Results:**

We measured total protein expression in isolated segments of jejunum using western blot from the following six groups of mice; intact or OVX with; 600G, 10E2 or control. The proteins of interest were: Akt, p-Akt, p-PDK1, p-PTEN, p-c-Raf, p-GSK-3β, rap-1 and ERK1/2. All blots were normalized to GAPDH levels (n = 6–18/group).

**Conclusion:**

These data suggest that the presence of the endogenous sex steroid, estrogen, modifies the intracellular signaling pathway required to mediate Cl^−^ secretion when the intestine is exposed to exogenous 600G or E2. These studies may have relevance for designing pharmacological tools for women with intestinal chloride secretory dysfunctions.

## Introduction

In murine jejunum, secretion of chloride, Cl^−^, from the crypts is widely known to involve the following: the bumetanide-sensitive Na^+^/K^+^/2Cl^−^ co-transporter supporting Cl^−^ entry into the cells, basolateral K^+^ channels enabling the recycling of K^+^ across the basolateral membrane and providing a driving force for Cl^−^ exit across the apical membrane, the Na^+^/K^+^ APTase serving to maintain Na^+^ and K^+^ concentrations gradients, and the cystic fibrosis conductance regulatory protein, the CFTR chloride channel, providing the major route for chloride exit across the apical membrane.

Flavonoids (such as genistein) are found naturally in soy and plants and are digested in an average daily diet. Genistein is structurally similar to estrogen [1, 2], and there is evidence to indicate that genistein binds to estrogen receptors, ERα and ERβ [3, 4], with comparable binding affinities to 17β-estradiol [5]. The ability of genistein to stimulate CFTR channel activity in isolated cells [6–8] or intact isolated tissues is well known [9–13], and genistein can stimulate both wild-type (Wt)-CFTR [14], and the most common CF disease-associated mutation, ΔF508-CFTR [15, 16].

We have previously described the effects of 1-week of daily subcutaneous, sc. injections of: genistein 600 mg/kg body weight/day (600G), vehicle control (0 mg/kg body weight/day, 0G), or estradiol 10 mg/kg body weight/day (10E2), on jejunum epithelial anion secretion from intact and ovariectomized (OVX) female mice [17]. Those studies demonstrated that intact and OVX mice responded similarly to 600G (I_sc_ was significantly increased in both groups), however, 10E2 only increased I_sc_ in the OVX mice (and was without effect in intact females). An advantage of the use of daily s.c. injections route of genistein administration versus diet, is that it can yield less variable and more sustained elevations in intestinal anion secretion, as we have shown previously [11, 17].

Here, we aimed to further understand genistein and estradiol’s mechanism(s) of action on jejunum Cl^−^ secretion in intact and OVX female mice, following treatment for 1-week with daily subcutaneous injection of 600G, 0G or 10E2, by determining their effects on several key intracellular signaling proteins. Improved understanding of the mechanism(s) of action of both genistein and estradiol on intestinal function may assist in the development of future selectively targeted pharmaceutical tools to mediate increases in intestinal transepithelial chloride secretion.

## Materials and Methods

### Mice

Female C57BL/6J intact or ovariectomized (OVX) mice were purchased from Jackson Laboratory (Bar Harbor, ME) at 4–6 weeks of age and housed in an animal care facility with 12:12-hour light-dark cycle. Mice consumed food and water ad libitum. Body weight and general health were monitored biweekly. Mice were fed a casein based genistein-free diet throughout the study and randomly assigned to one of the following injection groups administered each for a period of 1-week, with the dose administered once/daily via subcutaneous injection; 600G (600 mg genistein/kg body weight/day for 1-week), 0G (0 mg genistein/kg body weight/day for 1-week– genistein free), 10E2 (10 mg estradiol/kg body weight/day for 1-week).

At the end of the injection study period, mice were asphyxiated in an atmosphere of 100% CO_2_, followed by surgical thoracotomy to induce pneumothorax. Animal care and treatments were conducted in accordance with established guidelines and all protocols were approved by Midwestern University IACUC.

### Diet

The casein-based genistein-free diet was purchased from Dyets Inc (Bethlehem, PA) and had an estimated energy content of 16.28 kJ/g. Diet composition is described previously in Al-Nakkash et al [11]. Diet was assured genistein-free by Dyets Inc. and moreover, our past confirmatory measures have indicated that serum genistein levels are non-detectable with the genistein-free diet [17].

### Bioelectric measurement of intestinal secretion

Via an abdominal incision, ~5 cm of mid-jejunum was removed and placed in ice-cold oxygenated Krebs bicarbonate ringer (KBR). Each mouse yielded 2–3 jejunum pieces, isolated as described previously [17, 18]. Jejunum sections mounted in the Using chambers had 0.3 cm^2^ exposed surface area. Transepithelial short circuit current (I_sc_, μA/cm^2^) was measured via an automatic voltage clamp (VCC-600, Physiologic Instruments, San Diego, CA) and the experimental conditions and methods were as previously described [17, 18]. Intestinal tissue pieces were maintained in 1 μM indomethacin (minimizing tissue exposure to endogenously generated prostanoids due to manipulation and mounting of the tissue. Glucose (10 mM) was added to the serosal KBR bath and mannitol (10 mM) substituted for glucose in the mucosal KBR bath, to avoid an inward current due to Na^+^-coupled glucose transport. Once mounted, the serosal side was exposed to tetrodotoxin (0.1 μM), minimizing variations in intrinsic intestine neural tone. Intrinsic neural tone limits the absorptive capacity of the murine mucosa (decreased I_sc_ denotes neural block). *Experimental protocols:* Tissues were exposed to KBR (20 min) and steady-state basal I_sc_ measured at that time. Glucose (10 mM, mucosal) was added at the end of each experiment to stimulate Na^−^-coupled glucose transport and assess tissue viability (as denoted by > 10% increase in I_sc_). Tissues failing to respond to glucose within this parameter were discarded. *Solution:* Cl^−^-containing KBR contained the following (in mM): 115 NaCl, 25 NaHCO_3_, 5 KCl, 1.2 MgCl_2_ and 1.2 CaCl_2_, pH 7.4.

### Total protein expression utilizing western blot

At collection, segments of cleaned jejuna were immediately snap frozen in liquid nitrogen and stored at −80°C. Jejuna were later prepared for western blot analysis by homogenization. The western blot protocol was similar to that described previously [17]. Briefly, samples were analyzed for protein content, and ran on NuPAGE 4–12% Tris-Glycine gels (Thermo Fisher Scientific, Waltham, MA, USA) at 150 V for ~ 1.5 hours. Transfer was for 2 hours at 30 V on ice. Blots were incubated with primary antibody (all at 1:1000 dilution) to Akt, p-Akt, p-PDK1, p-PTEN, p-c-Raf, p-GSK-3β, p-p44/42 MAPK (Erk1/2), Rap1A overnight at 4°C. After washing, blots were incubated with secondary antibody, anti-rabbit immunoglobulin (IgG) (H+L) Dylight 800 Conjugate (1:15,000), for 1 hour at room temperature. To re-probe for glyceraldehyde 3-phosphate dehydrogenase (GAPDH), blots were incubated with anti-GAPDH primary antibody (1:4000) for 1 hour at room temperature. Blots were washed and then re-incubated with an anti-mouse (IgG) (H+L) Dylight 680 Conjugate (1:15,000), for 1 hour at room temperature. Images of the membranes were taken, with all protein normalized to GAPDH. Band density was analyzed using the Odyssey CLx infrared imaging system (LiCOR Biosciences, Inc., Lincoln, NE,USA) and Image Studio Software (LI-COR Biosciences, Inc.).

### Chemicals

Forskolin was purchased from Calbiochem (San Diego, CA). The following western blot antibodies: Akt, p-Akt, p-PDK1, p-PTEN, p-c-Raf, p-GSK-3β, p-p44/42 MAPK (Erk1/2), Rap1A, were all purchased from Cell Signaling Technology, Inc., (Danvers, MA). Secondary antibody, anti-rabbit immunoglobulin (IgG) (H+L) Dylight 800 Conjugate and anti-mouse (IgG) (H+L) Dylight 680 Conjugate was purchased from Thermo Fisher Scientific, Inc. (Waltham, MA). Anti-GAPDH primary antibody and all other chemicals were obtained from Sigma Aldrich (St. Louis, MO).

### Statistics

Data are expressed as mean ± SEM. Numbers in parentheses are numbers of tissues used from separate individual mice. One-way ANOVA with Neuman-Keul’s multiple comparison test or t-tests were performed using GraphPad (San Diego, CA). P < 0.05 was considered statistically significant.

## Results

### Effect of genistein and estradiol on basal Isc in jejunum

**Fig. 1:**
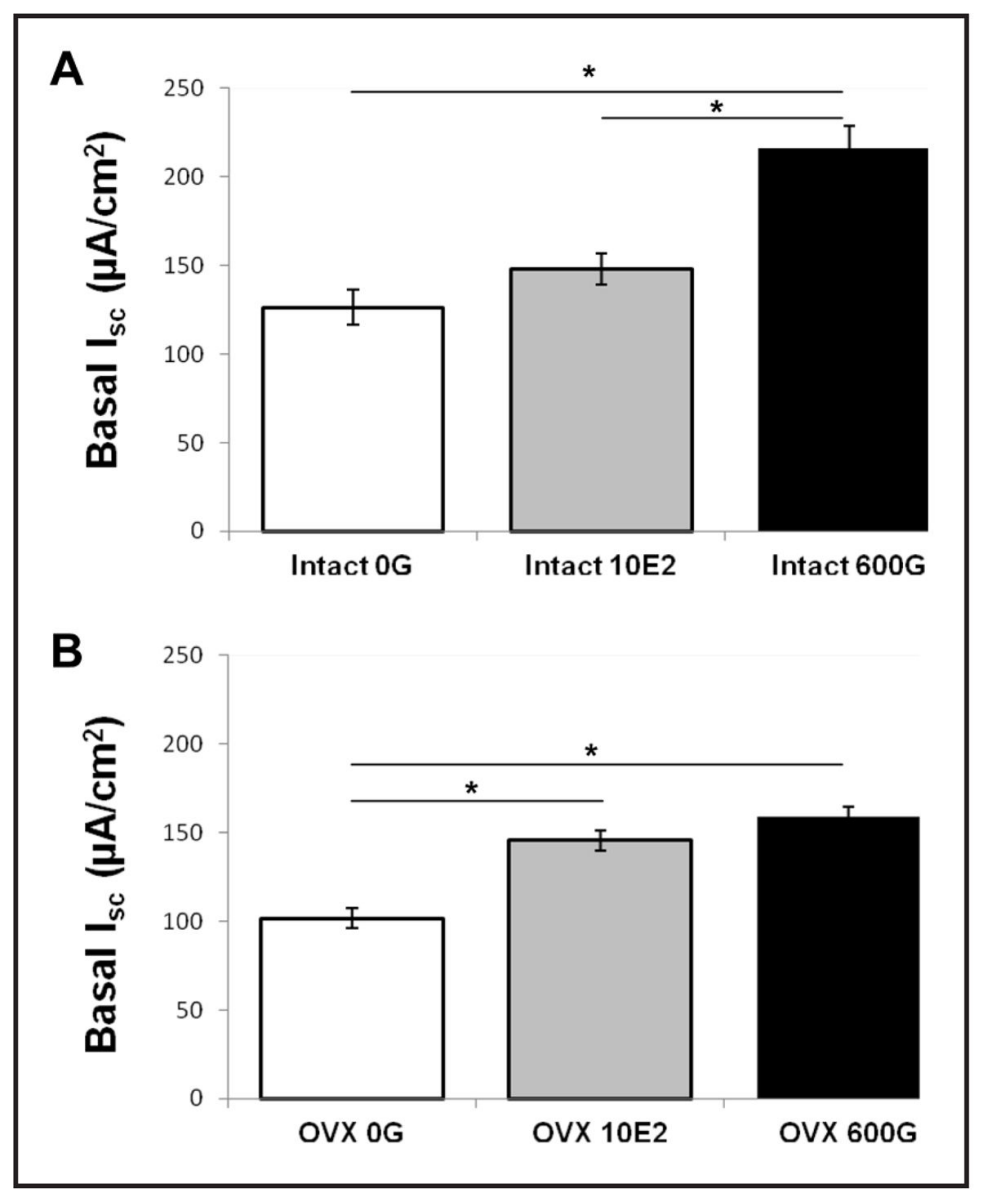
Effect of genistein (600G) and estradiol (10E2) on average basal I_sc_ in jejunum from intact and OVX female mice. A. Comparison of the basal I_sc_ from intact female mice injected for 1-week with either 600G (solid bar, n = 15), 10E2 (gray bar, n = 8), or 0G (open bar, n = 8). B. Comparison of the basal I_sc_ from OVX female mice injected for 1-week with either 600G (solid bar, n = 14), 10E2 (gray bar, n = 10), or 0G (open bar, n = 12). Values are mean ± SEM, * denotes significant difference from 0G, *P* < 0.05.

We have previously compared the effect of genistein (600G) and estradiol (10E2) (administered via daily subcutaneous injections for 1-week) on basal I_sc_ in jejunum from intact and OVX female mice [18]. In intact females, basal I_sc_ was significantly increased with 600G (1.7-fold increase compared to 0G controls), but unchanged by 10E2. In OVX females, basal I_sc_ was significantly increased by both 600G (1.6-fold increase compared to 0G controls) and 10E2 (1.5-fold increase compared to 0G controls) (Fig. 1). These data suggest that following the loss of endogenously produced estrogen via orchiectomy (OVX), both 10E2 and 600G act similarly and both cause an increase in the basal jejunum I_sc_. On the other hand, in the presence of endogenously produced estrogen, in intact female mice, then 10E2 and 600G have differing actions on basal I_sc_.

### Effect of genistein and estradiol on intestinal protein expression

**Fig. 2:**
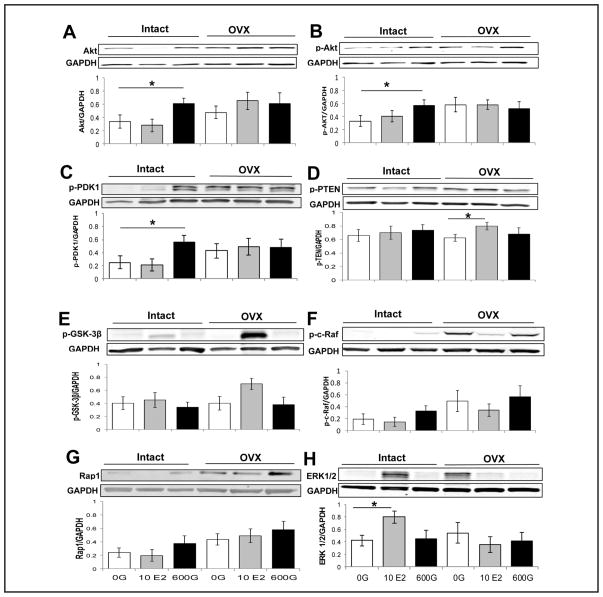
Effect of genistein or estradiol treatment on total Akt, p-Akt, p-PDK1, P-PTEN, p-GSK-3β and p-c-Raf, Rap1, and ERK1/2 protein expression in intact or ovariectomized female murine jejunum. A. Akt. Average Akt expression in female mice, following exposure to DMSO control (0G), estradiol (10E2), or genistein (600G), is shown normalized to GAPDH (n = 8–12). Representative western blot is shown above. Akt and GAPDH bands were observed at ~60 KDa and 37 KDa respectively. B. p-Akt. Average p-Akt expression in female mice, following exposure to DMSO control (0G), estradiol (10E2), or genistein (600G), is shown normalized to GAPDH (n = 9–12). Representative western blot is shown above. p-Akt and GAPDH bands were observed at ~60 KDa and 37 KDa respectively. C. p-PDK1. Average p-PDK1 expression in female mice, following exposure to DMSO control (0G), estradiol (10E2), or genistein (600G), is shown normalized to GAPDH (n = 9–11). Representative western blot is shown above. p-PDK1 and GAPDH bands were observed at ~58–68 KDa and 37 KDa respectively. D. p-PTEN. Average p-PTEN expression in female mice, following exposure to DMSO control (0G), estradiol (10E2), or genistein (600G), is shown normalized to GAPDH (n = 9–11). Representative western blot is shown above. p-PTEN and GAPDH bands were observed at ~54 KDa and 37 KDa respectively. E. p-GSK-3β. Average p-GSK-3β expression in female mice, following exposure to DMSO control (0G), estradiol (10E2), or genistein (600G), is shown normalized to GAPDH (n = 11–12). Representative western blot is shown above. p-GSK-3β and GAPDH bands were observed at ~46 KDa and 37 KDa respectively. F. p-c-Raf. Average p-c-Raf expression in female mice, following exposure to DMSO control (0G), estradiol (10E2), or genistein (600G), is shown normalized to GAPDH (n = 9–11). Representative western blot is shown above. p-c-Raf and GAPDH bands were observed at ~74 KDa and 37 KDa respectively. G. Rap1. Average Rap1 expression in female mice, following exposure to DMSO control (0G), estradiol (10E2), or genistein (600G), is shown normalized to GAPDH (n = 10–11). Representative western blot is shown above. Rap1 and GAPDH bands were observed at ~21 KDa and 37 KDa respectively. H. ERK1/2. Average ERK1/2 expression in female mice, following exposure to DMSO control (0G), estradiol (10E2), or genistein (600G), is shown normalized to GAPDH (n = 6–8). Representative western blot is shown above. ERK1/2 and GAPDH bands were observed at ~44 KDa and 37 KDa respectively.

We examined the effect of 600G and 10E2 on several key signaling pathway proteins to better understand their mechanism(s) of action. Total expression of Akt (a downtstream effector of phosphatidylinositol-3-kinase, PI3K), normalized to GAPDH was significantly increased in intact 600G (0.64 ± 0.08, n = 11, P < 0.05) compared to intact controls, 0G (0.35 ± 0.13, n = 11, Fig. 2A). There was no change in total expression of Akt in any of the other groups. Total expression of phosphorylation of Akt (p-Akt), normalized to GAPDH was significantly increased in intact 600G (0.52 ± 0.18, n = 12, P < 0.05) compared to intact controls, 0G (0.26 ± 0.06, n = 11, Fig. 2B). There was no change in total expression of p-Akt in any of the other groups. Total expression of p-PDK1 normalized to GAPDH was significantly increased in intact 600G (0.62 ± 0.10, n = 11, P < 0.05) compared to intact controls, 0G (0.26 ± 0.11, n = 10, Fig. 2C). There was no change in total expression of p-PDK1 in any of the other groups. Total expression of p-PTEN (a negative regulator of the PI3K/Akt pthway) normalized to GAPDH was significantly increased in OVX 10E2 (0.80 ± 0.06, n = 9, P < 0.05) compared to OVX controls, 0G (0.62 ± 0.05, n = 9, Fig. 2D). There was no change in total expression of p-PTEN (an activator of Akt) in any of the other groups. Total expression of p-GSK-3β (inactivated by Akt) normalized to GAPDH was significantly increased in OVX 10E2 (0.72 ± 0.10, n = 12, P < 0.05) compared to OVX controls, 0G (0.43 ± 0.34, n = 11, Fig. 2E). There was no change in total expression of p-GSK-3β in any of the other groups.

Total expression of either p-c-Raf or Rap1 normalized to GAPDH was unchanged by 600G or 10E2 in intact and OVX groups, Fig. 2F and 2G). Both p-c-Raf and Rap-1 are upstream kinase regulators/activators of ERK1/2 and thus the mitogen activated protein kinase cascade (MAPK). In addition, Rap1 is a direct downstream effector of EPAC1, and the Raf-ERK1/2 pathway can be activated by G-protein coupled receptors). Total expression of ERK1/2 normalized to GAPDH was significantly increased in intact 10E2 (0.82 ± 0.10, n = 8, P < 0.05) compared to intact controls, 0G (0.45 ± 0.10, n = 8, Fig. 2H). There was no change in total expression of ERK1/2 in any of the other groups.

## Discussion

In a previous study we described the effects of 1-week of daily subcutaneous, sc. injections of: genistein (600G), or estradiol (10E2), on jejunum epithelial anion secretion from intact and ovariectomized (OVX) female mice [17]. We demonstrated that intact and OVX mice responded similarly to 600G (basal I_sc_ was significantly increased in both groups), however, 10E2 increased I_sc_ only in the OVX mice (i.e. 10E2 had no effect in intact female mice). In this study, we aimed to provide mechanistic information regarding the signaling pathways involved with 600G and 10E2.

Consumption of naturally occurring dietary phytoestrogens, such as genistein, can result in micromolar serum genistein concentrations in humans [19]. We have previously shown that daily sc. administration of genistein (600G) for a period of 1- or 2-weeks in mice induces significant increases in Cl^−^ secretion (~80–86 μA/cm^2^) in freshly isolated jejuna segments, with concomitant increases in serum genistein levels (~4–8 μM) [17].

**Fig. 3:**
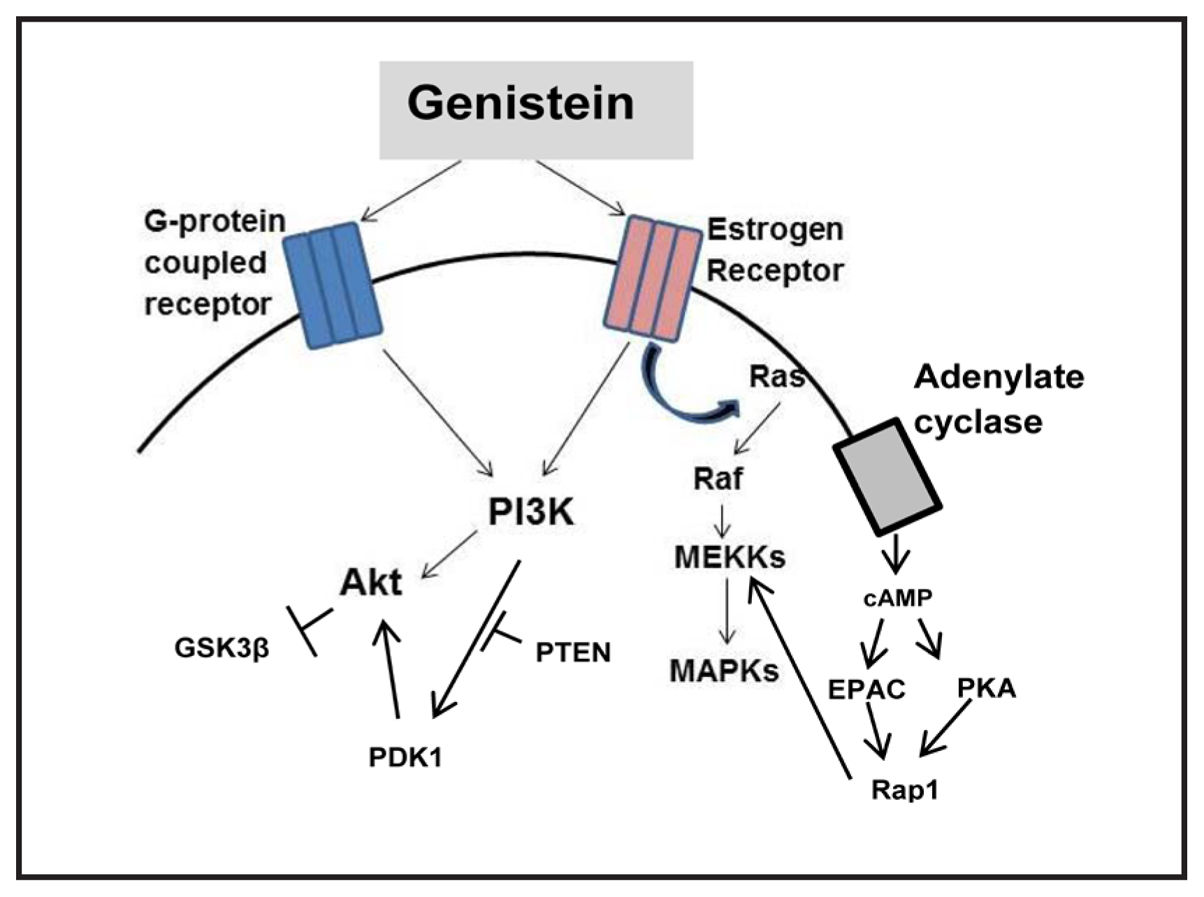
Signaling pathway map. A proposed schematic map of the signaling pathways through which either genistein or estradiol may mediate their effects.

The physiological roles that estradiol (either endogenous or exogenous) or genistein may play on intestinal function remains relatively ambiguous. Thus, this study aimed to determine the intracellular signaling pathways which are responsible for mediating the stimulatory effects on basal I_sc_, in intact or ovariectomized female mice of genistein and estradiol. We evaluated the involvement of key intracellular signaling pathways, thought to mediate G-protein coupled receptor-activation, estrogen receptor-activation, or adenylate cyclase (Fig. 3). It is widely postulated that activation of G protein coupled receptors and E2 receptors can mediate intracellular signaling pathways involving PI3K and Akt, and that E2 receptor activation may also mediate it’s effects via a pathway involving Ras-Raf and ERK1/2. Moreover, activation of adenylate cyclase can mediate intracellular signaling pathways involving cAMP, PKA, and EPAC which may also play a role in further stimulating ERK1/2. Thus, there are several potential pathways that may well be activated by the presence of either 600G or E2.

Genistein has previously been shown to mediate its effects on duodenal HCO_3_^−^ secretion through an estrogen receptor PI3K-dependent pathway [20]. In human colonic segments, genistein has been shown to rapidly and reversibly inhibit carbachol-induced colonic activity, via ERβ and utilizing p38/mitogen activated protein kinase mediated induction of nitric oxide [21]. Acute application of genistein has been shown to stimulate electrogenic Cl^−^ secretion across male murine jejunum [10]. Literature evidence and work from our laboratory has demonstrated that genistein acts on CFTR *in vitro* [14, 22], either by increasing channel activity or channel localization, or both [17, 23]. We have recently shown that genistein-mediated increases in jejunum basal I_sc_ can be attributed to a 5% concomitant increase in CFTR localization to the plasma membrane in intact males, yet this effect was not observed in intact females [17]. Thus, sex-dependent mechanism(s) are likely. Genistein has also been shown to up-regulate expression of genes either through the estrogen response element-dependent mechanisms or through estrogen response element-independent mechanisms. For instance, in Caco-2 human intestinal cells, genistein increases metallothionein expression which was shown to persist for at least 24 hours following removal of genistein from the cell culture medium [24, 25]. Estrogen receptors may also play a role in mediating the effects of genistein within the intestine, given the expression of both ERα and ERβ [26–29]. Evidence for interactions of genistein with ER’s comes from other work demonstrating that genistein modulates bone remodeling in osteoblastic cells, via ER’s, involving regulation of target gene expression [30]. Genistein has also been shown to be a ligand for gene expression regulation by either ERα or ERβ [31].

Adenylate cyclase (and cAMP) are well known key regulators of secretory ion transport across intestinal epithelial cell systems [32, 33]. Indeed, we have previously shown that genistein appeared to mediate stimulatory effects on jejunal I_sc_ in intact females via an adenylate cyclase-dependent mechanism [18].

Female sex hormones have also been described to play a contested role in the regulation of intestinal transport. Singh et al. [34] has shown that 17β-estradiol (E2) inhibits forskolin-stimulated chloride secretion across T84 epithelial cell monolayers. In distal colonic crypts from female rats, 17β-estradiol-mediated effects are thought to be mediated by inhibition of basolateral K^+^ channels, KCNQ1, via PKCδ- and PKA-dependent pathways [35–37]. E2 effects have also been linked to non-genomic inhibition of female rat colonic Cl^−^ secretion mediated via Ca^2+^ and PKC activation [38], likely mediated via inhibition of basolateral K_Ca_ and/or CFTR channel activity. In addition, progesterone and estradiol have been shown to inhibit CFTR-mediated ion transport in PANC-1 pancreatic epithelial cells [39], and in T84 intestinal cells [40]. Anti-secretory responses to estrogen may be characteristic of whole body salt and water retention during specific parts (specifically during high estrogen states) of the menstrual cycle, for instance right before ovulation in pre-menopausal women [41].

Interestingly, E2 has been shown to increase jejunal cellular proliferation in intact ewes, and to conversely decrease jejunal cellular proliferation in OVX ewes [42]. Given those proliferation data, one may expect that E2 would therefore increase I_sc_ in intact mice, and that conversely E2 may have no effect on I_sc_ in OVX mice, of course that prediction would likely be the case if intestinal cellular proliferation and intestinal secretion were directly related, and also, if ewes and mice behaved similarly. Of additional interest is the recent work by Kulkarni et al. [43], describing the effects of estrous cycle on genistein’s bioavailability; in intact females during times of proestrous (increased E2 levels) genistein levels were found to be low, whereas during metestrous (lowered E2 levels) genistein levels were found to be higher, OVX rats were similar to metestrous intact rats, and OVX+E2 rats had reduced genistein bioavailability. Given those bioavailability studies, our elevated basal I_sc_ data in the OVX mice injected with 600G, are not surprising (i.e. OVX animals being able to maintain increased genistein levels).

The data from this study demonstrates the following; (1) 10E2 does not increase basal I_sc_ in intact female mice, yet there is an increase in ERK1/2 expression, presumably via concomitant changes in Ras-Raf (i.e. activation of ER signaling), since there was no effect on expression of Rap1. (2) 600G increases basal I_sc_ in intact female mice, via increases in p-PDK1 and Akt expression, seemingly mediated by (either, or both) an action of genistein on G-protein coupled receptors and/or ER’s. This would fit with our previous findings demonstrating that 600G increases basal I_sc_ I intact females via stimulation of adenylate cyclase [18]. We suggest that involvement of G-protein coupled receptors is key for facilitating genistein-mediated increases in basal I_sc_ in intact mice, and likely there is no involvements of ER’s since our past work has demonstrated no change in ERβ expression with genistein treatment, and a significant genistein-mediated decrease in ERα expression [18] (3) 10E2 increases basal I_sc_ in OVX female mice, with associated increased expression of p-GSK-3β and p-PTEN. (4) 600G increases basal I_sc_ in OVX female mice, however, we did not measure notable changes in expression of any proteins examined (although there were trends for elevated expression of Rap1 and p-PDK1).

This work furthers our understanding of the effect and mechanism(s) of action of genistein and estradiol on jejuna I_sc,_ in a murine model, and it may be of benefit clinically in the evaluation and treatment of pre- and post-menopausal women with intestinal secretory dysfunction.
